# The role of purity and frequency in the classification of perimenstrual headache

**DOI:** 10.1186/s12883-023-03268-6

**Published:** 2023-06-06

**Authors:** Weinan Na, Hua Liu, Yang Liu, Xiaolin Wang, Shengyuan Yu

**Affiliations:** 1 Department of Neurology, the First Medical Center, Chinese PLA General Hospital, Beijing, People’s Republic of China; 2 School of Medicine, Nankai University, Tianjin, People’s Republic of China

**Keywords:** Menstruation, Headache, Nurse, Frequency, Purity

## Abstract

**Background:**

Among all menstruation-associated headaches, only menstrual migraine has classification criteria in the International Classification of Headache Disorders 3rd edition (ICHD-3). Other menstruation-associated headaches are not generally described. The ICHD-3 classifies menstrual migraine according to headache type, timing(on days -2 to +3 of menstruation), frequency (whether headache occurs in at least two out of three menstrual cycles), and purity(whether headache occurs at other times of the menstrual cycle), and provides a reference for research on menstruation-associated headache. However, the role of frequency and purity in the classification of menstruation- associated headache is not clear Moreover, the potential risk factors for high-frequency and pure headaches have not been explored.

**Methods:**

The study was a secondary analysis of an epidemiological survey on menstrual migraine among nurses. Among nurses who had a headache on days -2 to +3 of menstruation, headache frequency, purity, and type were described. High-frequency vs. low-frequency and pure vs. impure headache were compared according to headache features, demographics, occupation-related factors, menstruation-related factors, and lifestyle factors.

**Results:**

Of all respondents, 254(18.3%) nurses who had headaches on days -2 to +3 of menstruation were included in the study. In the 254 nurses with perimenstrual headache, the proportions of migraine, tension type headache (TTH), high-frequency headache, and pure headache were 24.4%, 26.4%, 39.0%, and 42.1%, respectively. High-frequency and impure perimenstrual headache was more severe and similar to migraine. High-frequency headache was associated with more perimenstrual extremity swelling and generalized pain. Other variables were not significantly different between the groups.

**Conclusions:**

Headache except for menstrual migraine accounts for a certain proportion of menstruation-associated headache and should not be ignored in research. Headache frequency and purity are related to headache type and should be equally considered in the classification of menstruation- associated headache. Perimenstrual extremity swelling and generalized pain are potential indicators of high-frequency perimenstrual headache.

**Trial registration:**

ChiCTR2200056429

**Supplementary Information:**

The online version contains supplementary material available at 10.1186/s12883-023-03268-6.

## Background

Menstruation-associated headache is a common chief complaint in gynecology and neurology clinics. Menstruation-associated headache has been studied as a part of premenstrual syndrome (PMS) in the gynecology field [1, 2], but has not been thoroughly described or strictly classified according to timing, frequency, or type as in the neurology field. Among all menstruation-associated headaches, only menstrual migraine hasclassification criteria since the International Classification of Headache Disorders 2nd edition (ICHD-2) [3] was released in 2004. The criteria identified menstrual migraine according to headache type (migraine), timing (on days -2 to +3 of menstruation) and relationship to menstruation. The menstruation-headache relationship includes headache frequency (whether headache occurs in at least two out of three menstrual cycles) and headache purity (whether headache occurs at other times of the menstrual cycle).

The criteria for menstrual migraine provide a reference for research on menstruation-associated headache but simultaneously lead to ignorance of other menstruation-associated headaches except for menstrual migraine. Whether these headaches should be treated as a part of PMS or as an independent headache disorder entity is controversial [4, 5]. Menstruation-associated headache includes not only migraines, but also tension type headaches (TTHs) and other types [4, 6, 7], but these types are ignored in research and have not been reasonably classified or identified. There have been several studies [6–9] focusing on menstrual TTH but we still pay insufficient attention to menstrual headache beyond menstrual migraine.

Given the fixed headache timing (on days -2 to +3 of menstruation, referred to as perimenstrual headache in this article), whether headache frequency and purity are necessaryfor the classification of menstruation-associated headache in clinical research is still not clear because the difference between high-frequency and low-frequency headache or that between pure and impure headache has not been clarified.

This study aimed to propose an overall assessment of menstruation-associated headache. In individuals who had a headache on days -2 to +3 of menstruation, we compared high-frequency headache with low-frequency headache and pure headache with impure headache according to headache features, demographics, occupation-related factors, menstruation-related factors, perimenstrual symptoms, and lifestyle factors. The above comparison aimed to clarify the role of frequency and purity in the classification of menstruation- associated headache. Moreover, the comparison attempted to offer clues regarding risk factors for high-frequency and pure headache for which premenstrual prophylaxis may be cost-effective.

## Methods

The study was a secondary analysis of an epidemiological survey on menstrual migraine among nurses from multiple clinical centers of a large 3A hospital in Beijing. We randomly selected 30 clinical departments. All menstruating female nurses from the selected department were invited to participate in the survey. Participants completed a structured self-report questionnaire including four mandatory sections and one optional section. The former included demographic factors, occupation-related factors, lifestyle profile questions and menstrual features; the latter included headache features. If a respondent answered “yes” for the question “In the last year, have you had headache during the week before menstruation or during menstruation?”, then the headache feature section was completed, including questions about whether they have headaches at -2 to +3 of menstruation, whether they have headaches at other times of the menstrual cycle, and whether they had headaches at least two out of three cycles. Among all respondents, those who had headache on days -2 to +3 of menstruation were included and referred to as nurses with perimenstrual headache. To avoid chronic headache associated with menstruation by chance, respondents who reported a monthly headache days as greater than or equal to 15 days were excluded.

High-frequency perimenstrual headache referred to headache in at least two out of three menstrual cycles. Pure perimenstrual headache referred to no headache at other times except for -2 to +3 of menstruation. The comparison was conducted between high vs. low-frequency and pure vs. impure perimenstrual headache according to headache features, demographics, occupation-related factors, menstruation-related factors, and lifestyle factors.

The variables compared include headache features (duration, side, pain intensity, location, throbbing, accompanying symptoms, aggravation with activity, need for pain killers, and family history), demographics (age, BMI, ethnicity, marital status, constellation, and education), occupation-related factors (working years, department, title, number of night shift times per month, and number of night shift hours per month), menstruation-related factors (menarche age, menstruation regularity, menstruation length, and 27 other perimenstrual symptoms), and lifestyle factors(smoking, drinking, exercise, coffee consumption, tea consumption, sugary beverage consumption, skipping breakfast, difficulty falling asleep, dreaminess, early waking, daytime drowsiness, number of sleep hours per day, and number of sitting hours per day).

Non-normally distributed continuous variables are summarized as medians(IQRs). Categorical variables are summarized as numbers and percentages. The Mann–Whitney U test was used for comparisons of non-normally distributed continuous variables and ranked variables. The Chi-square test or Fisher’s exact test was used for comparisons of categorical variables. Statistical significance was set at *p* < 0.05 (two-tailed). All statistical analyses were performed with SPSS (23.0).

The study protocol was approved by the Ethics Committee of the Chinese PLA General Hospital, Beijing. All respondents provided written informed consent after a detailed explanation for study participation. The study was registered in the Chinese Clinical Trial Registry (ChiCTR2200056429).

As there are complicated terminology and definitions in this paper, the definitions are summarized as follows. Menstruation-associated headache refers to all headaches associated with menstruation in a wide sense without special qualifications regarding the relationship between headache and menstruation. Perimenstrual headache refers to headaches on days -2 to +3 of menstruation (the first day of bleeding as day 1 and the day before bleeding as day-1). High-frequency perimenstrual headache refers to headaches on days -2 to +3 of menstruation and at least two out of three menstrual cycles. Pure perimenstrual headache refers to headaches on days -2 to +3 of menstruation but not at other times of the menstrual cycle. Menstrual migraine refers to high-frequency migraine (the same as ICHD-2 and ICHD-3).

## Results

### Demographics of the study population

**Fig. 1:**
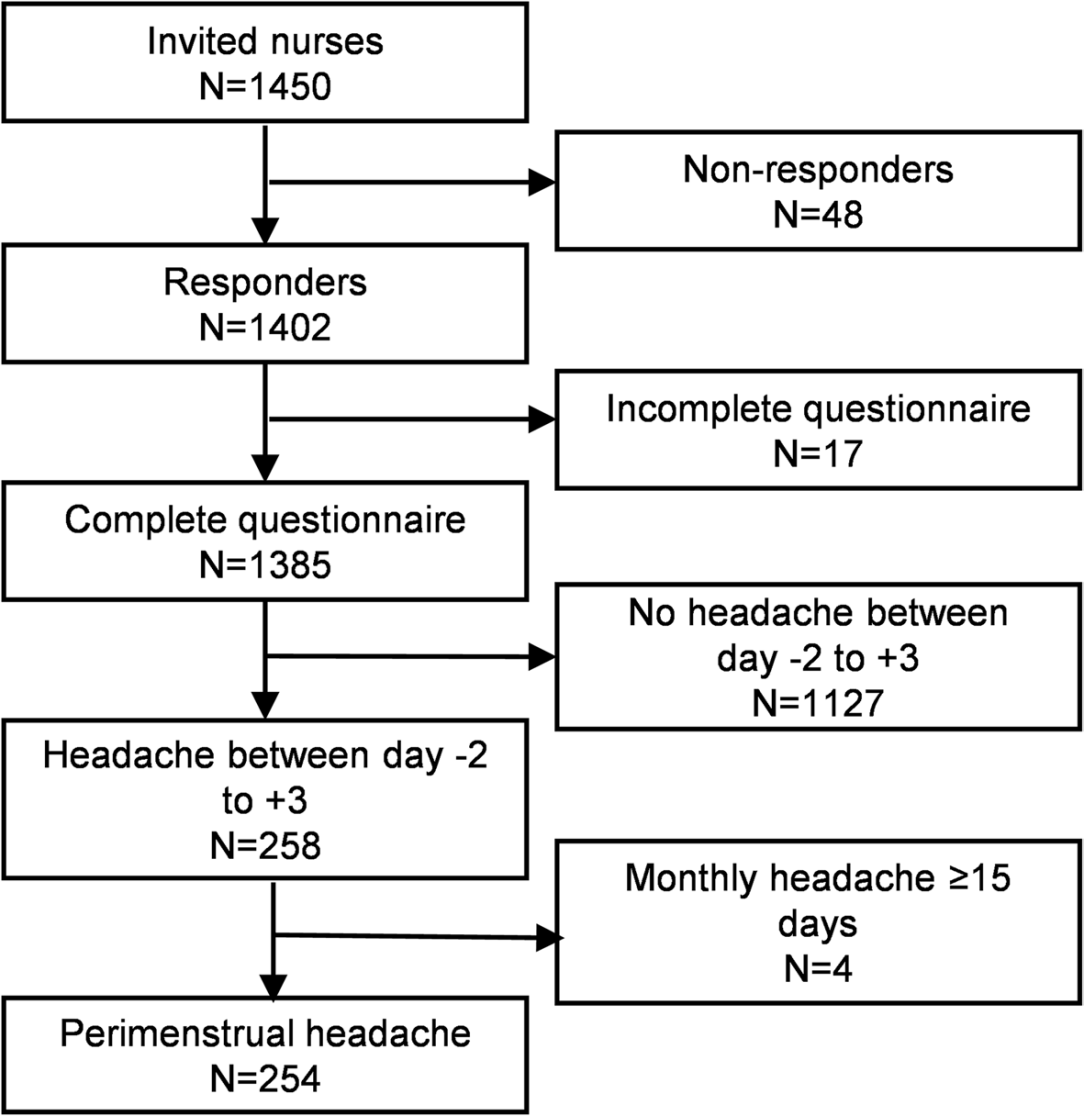
Flow chart of the study

Figure 1 shows the flowchart of the study. Of all respondents, 258(18.6%) nurses had a headache on days -2 to +3 of menstruation. After excluding 4 nurses who had a headache equal to or more than 15 days per month, 254(18.3%) nurses with perimenstrual headache were included in the study. Table 1 shows the demographics of the 254 nurses with perimenstrual headache. The mean age was 29 years (25, 34) and the mean working years is 7(3, 12).

**Table 1:** Demographics of the 254 nurses with perimenstrual headache

	N (%) or Median (IQR)
Age	29 (25, 34)
BMI	21.0 (19.5, 23.2)
Ethnicity	
Han	239 (94.1)
Not Han	15 (5.9)
Marital status	
Unmarried	131 (51.6)
Others	123 (48.4)
Education	
Below college	77 (30.3)
College and above	177 (69.7)
Working years	7 (3, 12)
Department	
Internal medicine	147 (57.9)
Surgery department	76 (29.2)
Others	31 (12.2)

### Classification and features of perimenstrual headache

**Fig. 2:**
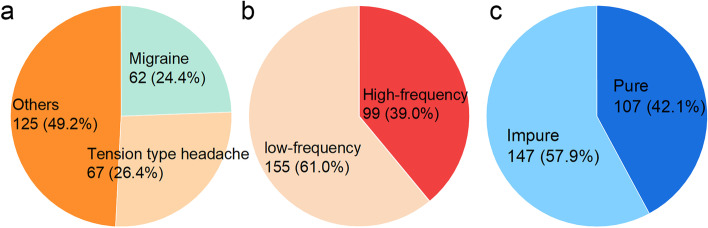
Classification of perimenstrual headache. Migraine and tension type headache (TTH) follows ICHD-3; High frequency: headache at least two out of three menstrual cycles; Pure: no headache at other times except for -2 to +3 of menstruation; Total = 254

Figure 2 shows the classification and features of perimenstrual headache. Of the 254 nurses with perimenstrual headache, the proportions of those with migraine, TTH, high-frequency headache, and pure headache were 24.4%, 26.4%, 39%, and 42.1%, respectively. The proportion of pure perimenstrual headache was not significantly different between the high and low-frequency headache groups(43.4% vs.41.3%, *p* = 0.736). Table 2 shows the crosstabulation of perimenstrual headache. The most common headache was low-frequency, impure, other types of headache (18.1%), and the least common headache was high-frequency pure TTH (4.7%). Of the 99 nurses with high-frequency headaches, 31.3%(31/99) met the criteria for menstrual migraine, and 68.7% is non-migraine headache. Specifically, 25.3%(25/99) of high-frequency headaches were TTHs, and 43.4%(43/99) were other types of headaches.

**Table 2:** Crosstab of perimenstrual headache

	N (%)			
	Migraine	TTH	Other types	Total
High-frequency				
Impure	17 (6.7)	13 (5.1)	26 (10.2)	56 (22.0)
Pure	14 (5.5)	12 (4.7)	17 (6.7)	43 (16.9)
Low-frequency				
Impure	18 (7.1)	27 (10.6)	46 (18.1)	91 (35.8)
Pure	13 (5.1)	15 (5.9)	36 (14.2)	64 (25.2)
Total	62 (24.4)	67 (26.4)	125 (49.2)	254 (100.0)

High frequency: headache at least two out of three menstrual cycles; Pure: no headache at other times except for -2 to +3 of menstruation; Migraine and tension type headache (TTH) follows ICHD-3

### Comparison between the high- and low-frequency perimenstrual headache groups

**Fig. 3:**
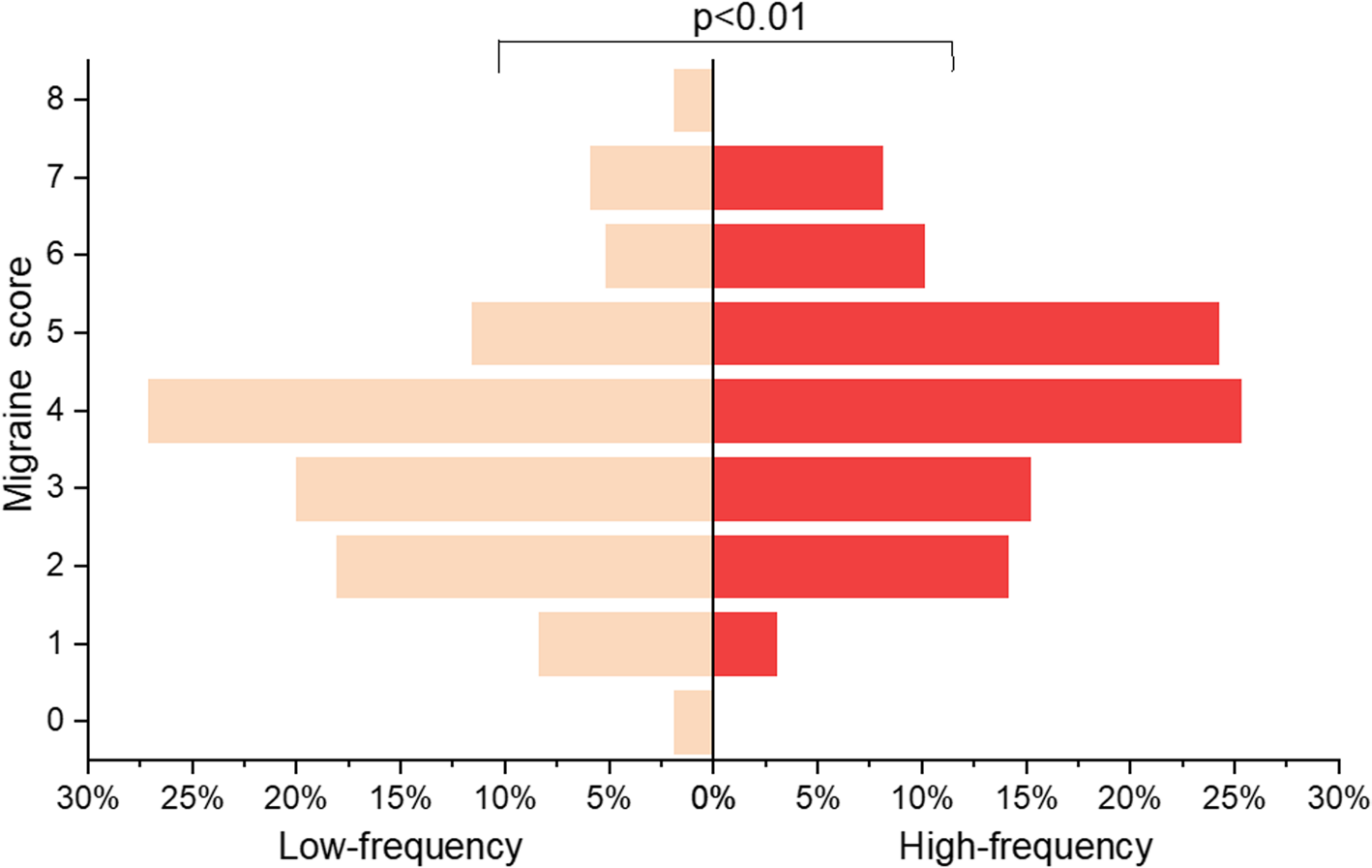
Comparison of migraine score between high-frequency and low-frequency perimenstrual headache; Migraine score: duration 4-72 h = 1, unilateral = 1, moderate to severe = 1, throbbing = 1, aggravation with activity = 1, nausea = 1, vomiting = 1, photophobia or phonophobia = 1; High frequency: headache at least two out of three menstrual cycles

Comparisons were made between the high- and low-frequency perimenstrual headache groups. Variables that were statistically significantly different between the two groups (headache features) are shown in Table 3. Compared with low-frequency headache, high-frequency headache was associated with a longer duration, more frontal pain, more pronounced phonophobia, higher pain intensity, more painkiller intake, early onset (Table 3), higher migraine scores (Fig. 3), more perimenstrual extremity swelling, and more perimenstrual generalized pain. Demographics, occupation-related, menstruation-related, and lifestyle factors were not significantly different between the two groups (Additional file 1).

**Table 3:** Comparison between high-frequency and low-frequency perimenstrual headache (variables with statistical difference)

	N (%) or Median (IQR)		*P*
	Low-frequency perimenstrual headache *N* = 155	High-frequency perimenstrual headache *N* = 99	
Headache duration			
Shorter than 4 h	45 (29.0)	12 (12.1)	0.002^a^**
Longer than 4 h	110 (71.0)	87 (87.9)	
Frontal			
No	108 (69.7)	56 (56.6)	0.033^a^*
Yes	47 (30.3)	43 (43.4)	
Phonophobia			
No	87 (56.1)	38 (38.4)	0.006^a^**
Yes	68 (43.9)	61 (61.6)	
Pain intensity^c^			
Mild	72 (46.5)	31 (31.3)	0.017^a^*
Moderate to severe	83 (53.5)	68 (68.7)	
Need pain killer			
No	135 (87.1)	72 (72.7)	0.004^a^**
Yes	20 (12.9)	27 (27.3)	
Onset of headache^d^			
Before day 1	42 (27.1)	41 (41.4)	0.037^a^*
On day 1	75 (48.4)	34 (34.3)	
After day 1	38 (24.5)	24 (24.2)	
Migraine			
No	124 (80.0)	68 (68.7)	0.041^a^*
Yes	31 (20.0)	31 (31.3)	
Migraine score^e^	4 (2,4)	4 (3,5)	0.003^b^**
Perimenstrual symptoms^f^			
Extremity Swelling	37 (23.9)	37 (37.4)	0.021^a^*
Generalized pain	55 (35.5)	53 (53.5)	0.005^a^**

^*^p < 0.05, **p < 0.01

^a^Chi-squire

^b^Mann-Whitney U test

^c^Pain intensity is evaluated by Visual Analogue Scale (VAS), mild (1–3), moderate (4–6), severe (7–10)

^d^Onset of headache defines the first day of bleeding as day 1 and the day before bleeding as day-1 (consisting with ICHD-3)

^e^Migraine score: duration 4-72 h = 1, unilateral = 1, Moderate to severe = 1, throbbing = 1, Aggravation with activity = 1, Nausea = 1, Vomiting = 1, Photophobia or Phonophobia = 1

^f^27 perimenstrual symptoms were surveyed in total, perimenstrual symptoms refers to whether had certain symptom between 7 days before menstruation and the end of menstruation in the last year

### Comparison between the pure and impure perimenstrual headache groups

**Fig. 4:**
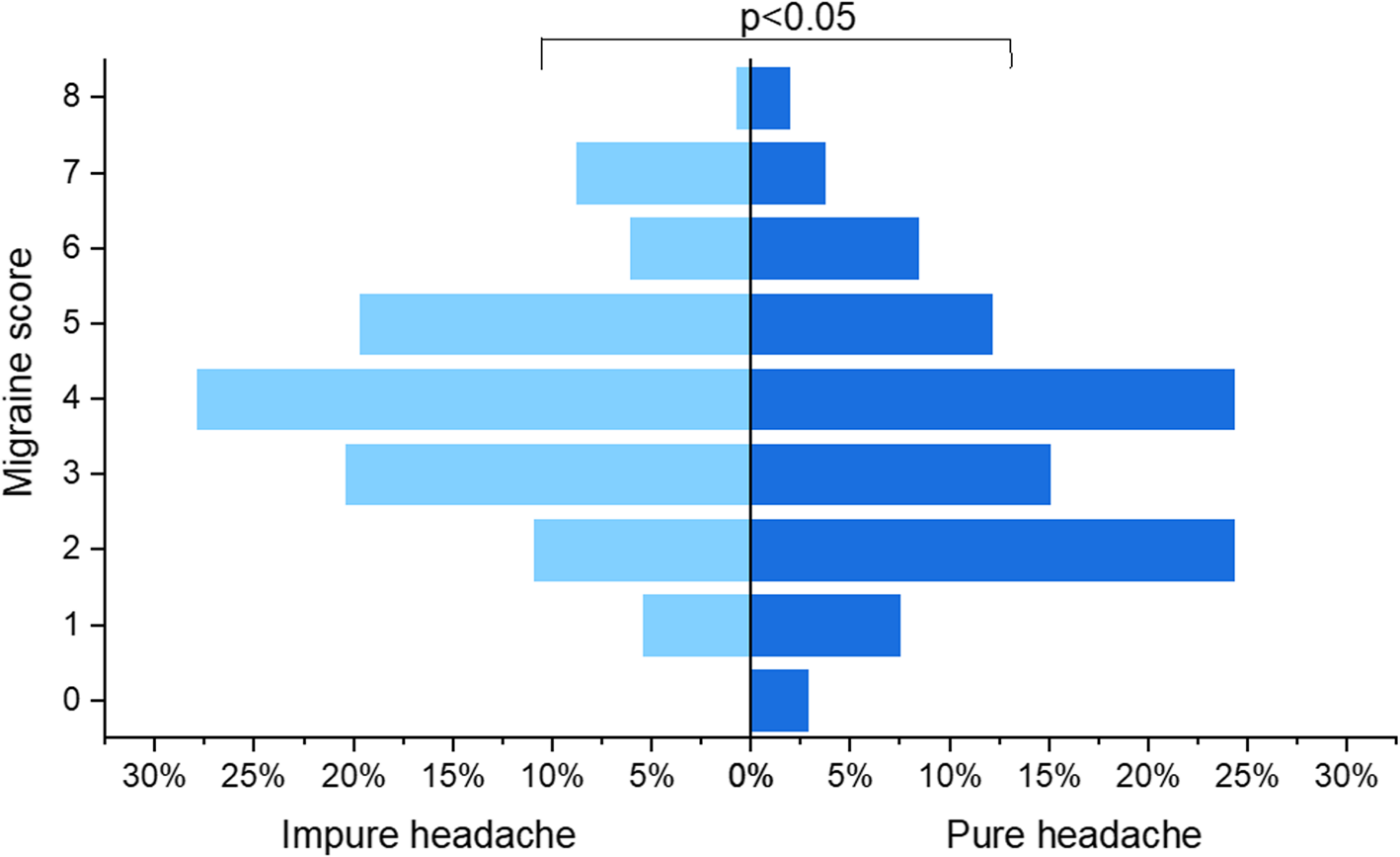
Comparison of migraine score between pure and impure perimenstrual headache. Migraine score: duration 4-72 h = 1, unilateral = 1, moderate to severe = 1, throbbing = 1, aggravation with activity = 1, nausea = 1, vomiting = 1, photophobia or phonophobia = 1; Pure: no headache at other times except for -2 to +3 of menstruation

Comparisons were conducted between the pure and impure perimenstrual headache groups. Variables with significant differencees (headache features) are shown in Table 4. Compared with pure perimenstrual headache, impure headache was associated with earlier onset, multiple locations, more pronounced phonophobia, higher pain intensity (Table 4), and higher migraine score (Fig. 4). Demographics, occupation-related, menstruation-related, and lifestyle factors were not statistically significantly different (Additional file 2).

**Table 4:** Comparison between pure and impure perimenstrual headache (variables with statistical difference)

	N(%) or Median(IQR)		*P*
	Impure perimenstrual headache *N* = 147	Pure perimenstrual headache *N* = 107	
Onset of headache^c^			
Before day 1	58(39.5)	25(23.4)	0.024^a^*
On day 1	58(39.5)	51(47.7)	
After day 1	31(21.1)	31(29.0)	
Location number^d^			
Single	61(41.5)	65(60.7)	0.002^a^**
Multiple	86(58.5)	42(39.3)	
Phonophobia			
No	65(44.2)	60(56.1)	0.062^a^*
Yes	82(55.8)	47(43.9)	
Pain intensity^e^			
Mild	51(34.7)	52(48.6)	0.026^a^*
Moderate to severe	96(65.3)	55(51.4)	
Migraine score^f^	4(3, 5)	4(2, 5)	0.012^b^*

^*^p < 0.05, **p < 0.01

^a^Chi-squire

^b^Mann-Whitney U test

^c^Day of onset of headache defines the first day of bleeding as day 1 and the day before bleeding as day-1 (consisting with ICHD-3)

^d^Location number defines the same location of the bilateral head as single, different location as multiple, representing the diversity of affected head location

^e^Pain intensity is evaluated by Visual Analogue Scale (VAS), mild (1–3), moderate (4–6), severe (7–10)

^f^Migraine score: duration 4-72 h = 1, unilateral = 1, moderate to severe = 1, throbbing = 1, aggravation with activity = 1, nausea = 1, vomiting = 1, photophobia or phonophobia = 1

## Discussion

In this study, we described an overall assessment of perimenstrual headache. Then we compared headache features, demographics, occupation-related factors, menstruation-related factors, and lifestyle factors between high-frequency vs. low-frequency and pure vs. impure perimenstrual headache groups. High-frequency perimenstrual headache and impure perimenstrual headache were approximate to migraine.

Menstrual migraine is a part of perimenstrual headache, but not the full part. Except for migraine type, TTH and other types of headaches account for a certain proportion of perimenstrual headache. In our study, 75.6% of perimenstrual headaches and 68.7% of high-frequency perimenstrual headaches were non-migraine headache. Moreover, the headache type relies on the description provided by a patient with a perimenstrual headache, which is not as precise as the description provided by a patient with chronic headache. Restricting criteria on headache type may let us ignore some headaches related to menstruation. A clinic-based study [4] on high-frequency perimenstrual headache in Serbia showed that 24% of headaches were non-migraine headaches, and this proportion was smaller than that of our research (68.7%). Their study included 50 patients from the clinic, which is a small sample size and their respondent composition may be different from that of our study based on the general population of a special occupation. Migraine may be more severe than non-migraine even at an equal headache frequency, leading to more doctor-seeking behavior.

Menstrual TTH is not included in the ICHD, but some researchers have paid attention to this potential special type of headache. Compared to migraine, TTH can also be triggered by menses [8, 10–12], be menstrual- associated [6], and even be more likely to occur exclusively during menstruation [13]. A clinic-based study [7] first defined menstrual TTH in 2007, which is consistent with the high-frequency TTH in our study. Their research showed that 21 of 165 (12.7%) patients whose headache was related to menstruation (the same definition as high-frequency perimenstrual headache in our study) had menstrual TTH. In our study, 9.8% (25/254) of perimenstrual headaches and 25.3% (25/99) of high-frequency perimenstrual headaches met the criteria for menstrual TTH. The different respondent compositions may explain the difference between the proportion in their study and ours. Another study on menstrual TTH focused on the proportion of menstrual TTH in all TTH. A cross-sectional study [6] among medical students showed that menstrual-related headache occurs in 6.3% of women with TTH. Both the proportion of menstrual TTH in TTH and that in menstrual headache is small. However, a small proportion does not deny the worthiness of study, but it means we should focus on a narrower population who truly has menstrual TTH. Maybe considering menstrual non-migraine headache as a whole part in research is a new research strategy.

Given the fixed timing of headache duringmenstruation (on days -2 to +3), the relationship between menstruation and headache can be described in two aspects: frequency and purity. High-frequency and pure perimenstrual headache can be regarded as a close menstruation-headache relationship. According to the criteria for pure menstrual migraine (PMM) and menstrually related migraine (MRM), the ICHD-3 emphasizes frequency more than purity when classifying migraine attack on days -2 to +3 of menstruation. Individuals who have a headache in less than two out of three menstrual cycles are classified as non-menstrual migraine even though they experience headache exclusively during menstruation. In our study, frequency and purity were associated with headache type, but the association seems contradictory. High-frequency perimenstrual headache is similar to migraine, but pure perimenstrual headache isnot. Moreover, headache purity and frequency are not associated with each other. Therefore, we propose that the frequency and purity of perimenstrual headache should be equally considered in the evaluation of the menstruation-headache relationship, except for the evaluation of headache type. Frequency and purity were associated with headache type but were not associated with demographics, occupation, or lifestyle. Compared with low-frequency headache, high-frequency headache is associated with more perimenstrual extremity swelling and generalized pain. These two symptoms may be indicators of high-frequency perimenstrual headache. The risk factors for close menstruation-headache relationship need further study because patient with these headaches may benefit from premenstrual prophylaxis.

In our research, the respondents were nurses, an occupation-specialized population mainly consisting of reproductive-age females. Previous studies [14, 15] focusing on headache in nursing staff suggest that this population is more likely to have primary headaches and that night shifts and associated sleep disorders play a particular role. Therefore, we collected information about department, title, and night shift to identify potential confounding factors. Although these occupation-related variables were not significantly different between the high-frequency vs. low-frequency and pure vs. impure headache groups, we still suggest a cautious extrapolation to the general population.

In our study, both kinds of menstrual headache included headaches occurring at other times of the menstrual cycle, which have led to a possible accidental association between menstruation and headache. We excluded individuals who had a headache more than or equal to 15 days per month to eliminate possible accidental associations. Moreover, in the ICHD criteria for menstrual migraine, this kind of “loose” association is allowed. Perhaps a group of headaches that occur only during menstruation should be focused on when the sample size is large enough to show the reality.

We do not have the information on the headache features of individuals who only have headaches outside of the perimenstrual perimenstrual period. Studies on migraine, TTH and other primary headache among nursing stuff have been done previously [14, 15] and we specifically focused on headaches that occur during the perimesntrual period in the current study.

## Conclusions

Headache except for menstrual migraine accounts for a certain proportion of menstruation-associated headache and should not be ignored in research. Headaches closely associated with menstruation include migraine, TTH, and other types. Headache frequency and purity are related to headache type and should be equally considered in the classification of menstruation-associated headache. High-frequency and impure headache is similar to migraine. Perimenstrual extremity swelling and generalized pain are potential indicators of high-frequency perimenstrual headache. The risk factors for close menstruation-headache relationship needs further study because individuals with these headache may benefit from premenstrual prophylaxis.

## Supplementary Information

**Additional file 1: Table S1.** Comparison between low-frequency and high-frequency perimenstrual headache (variables with no statistical difference).

**Additional file 2: Table S2.** Comparison between impure and pure perimenstrual headache (variables with no statistical difference).

## Data Availability

The data that support the findings of this study are available from the corresponding author on reasonable request.

## Abbreviations

ICHD: International Classification of Headache Disorders
TTH: Tension type headache
VAS: Visual Analogue Scale
