# Supplementary material for: The role of purity and frequency in the classification of perimenstrual headache
Source: BMC Neurol. 2023 Jun 6;23:218. doi: 10.1186/s12883-023-03268-6 (PMC10242800; doi:10.1186/s12883-023-03268-6)
Supplement: Supplementary file 1 — Additional file 1: Table S1. Comparison between low-frequency and high-frequency perimenstrual headache (variables with no statistical difference). [file 12883_2023_3268_MOESM1_ESM.pdf]

**TableS1** Comparison between low-frequency and high-frequency perimenstrual headache (variables with no statistical difference)

| | | N(%) OR Median(IQR) | | p |
|---|---|---|---|---|
| | | Low-frequency perimenstrual headache | High-frequency perimenstrual headache | |
| Headache feature | | | | |
| Headache side | Unilateral | 77(49.7) | 45(45.5) | 0.511[a] |
| | Bilateral | 78(50.3) | 54(54.5) | |
| Parietal | No | 105(67.7) | 63(63.6) | 0.500 [a] |
| | Yes | 50(32.3) | 36(36.4) | |
| Temporal | No | 49(31.6) | 33(33.3) | 0.775 [a] |
| | Yes | 106(68.4) | 66(66.7) | |
| Other location [d] | No | 97(62.6) | 62(62.6) | 0.944 [a] |
| | yes | 58(37.4) | 37(37.4) | |
| Location number [e] | Single | 76(49.0) | 50(50.5) | 0.819 [a] |
| | Multiple | 79(51.0) | 49(49.5) | |
| Throbbing headache | No | 95(61.3) | 57(57.6) | 0.556 [a] |
| | Yes | 60(38.7) | 42(42.4) | |
| Accompanying symptom | No | 38(24.5) | 16(16.2) | 0.112 [a] |
| | Yes | 117(75.5) | 83(83.8) | |
| Nausea | No | 82(52.9) | 47(47.5) | 0.399 [a] |
| | Yes | 73(47.1) | 52(52.5) | |
| Vomiting | No | 123(79.4) | 81(81.8) | 0.630 [a] |
| | Yes | 32(20.6) | 18(18.2) | |
| Photophobia | No | 138(89.0) | 83(83.8) | 0.230 [a] |
| | Yes | 17(11.0) | 16(16.2) | |
| Aggravation with activity | No | 97(62.6) | 67(67.7) | 0.408 [a] |
| | yes | 58(37.4) | 32(32.3) | |
| Family history | No | 119(76.8) | 72(72.7) | 0.466 [a] |
| | Yes | 36(23.2) | 27(27.3) | |
| Demographics | | | | |
| Age | | 30(24,34) | 29(25,35) | 0.549 [b] |
| BMI | | 21.2(19.5,23.1) | 20.6(19.3,23.4) | 0.369 [b] |
| Ethnicity | Han | 144(92.9) | 95(96.0) | 0.314 [a] |
| | Not Han | 11(7.1) | 4(4.0) | |
| Marital status | Unmarried | 79(51.0) | 52(52.5) | 0.809 [a] |
| | Others | 76(49.0) | 47(47.5) | |
| Constellation [f] | Yang | 78(50.3) | 52(52.5) | 0.723 [a] |
| | Yin | 77(49.7) | 47(47.5) | |
| Education | Below college | 46(29.7) | 31(31.3) | 0.782 [a] |
| | College and above | 109(70.3) | 68(68.7) | |
| Occupation | | | | |

| | | | | |
|---|---|---|---|---|
| Working years | | 7.0(3.0,11.0) | 7.0(4.0,13.0) | 0.632 [b] |
| Department | Internal medicine | 83(53.5) | 64(64.6) | 0.100 [a] |
| | Surgery department | 54(34.8) | 22(22.2) | |
| | others | 18(11.6) | 13(13.1) | |
| Title | Primary nurse | 48(31.0) | 28(28.3) | 0.760 [a] |
| | Senior nurse | 62(40.0) | 38(38.4) | |
| | Nurse in charge and above | 45(29.0) | 33(33.3) | |
| Night shift times per month | | 5.0(1.0,8.0) | 4.0(1.0,8.0) | 0.942 [b] |
| Night shift hours per month | | 27.0(4.0,60.0) | 14.0(4.0,48.0) | 0.657 [b] |
| Menstruation | | | | |
| Menarche age | | 13(13,14) | 13(12,14) | 0.207 [b] |
| Menstruation regularity[g] | Regular | 78(50.3) | 54(54.5) | 0.511 [a] |
| | Not regular | 77(49.7) | 45(45.5) | |
| Menstruation length[g] | Normal | 152(98.1) | 92(92.9) | 0.051 [c] |
| | Too short or too long | 3(1.9) | 7(7.1) | |
| Perimenstrual symptom number [h] | | 13(9,18) | 14(8,19) | 0.463 [b] |
| Perimenstrual symptoms | Irritability | 121(78.1) | 84(84.8) | 0.181 [a] |
| | Anxiety | 106(68.4) | 68(68.7) | 0.960 [a] |
| | Depressed mood | 76(49.0) | 53(53.5) | 0.484 [a] |
| | Fidgeting | 62(40.0) | 39(39.4) | 0.923 [a] |
| | Social withdrawl | 64(41.3) | 44(44.4) | 0.620 [a] |
| | Confusion | 74(47.7) | 42(42.4) | 0.407 [a] |
| | Low concentration | 102(65.8) | 59(59.6) | 0.316 [a] |
| | Somnolence | 69(44.5) | 45(45.5) | 0.883 [a] |
| | Insomnia | 63(40.6) | 47(47.5) | 0.284 [a] |
| | Breast tenderness | 115(74.2) | 75(75.8) | 0.779 [a] |
| | Dizziness | 93(60.0) | 64(64.6) | 0.457 [a] |
| | Nausea | 69(44.5) | 48(48.5) | 0.536 [a] |
| | Vomiting | 37(23.9) | 27(27.3) | 0.543 [a] |
| | Decreased appetite | 86(55.5) | 60(60.6) | 0.421 [a] |
| | Abdominal bloating | 94(60.6) | 63(63.6) | 0.632 [a] |
| | Constipation | 54(34.8) | 31(31.3) | 0.561 [a] |
| | Urgency to defecate | 53(34.2) | 31(31.3) | 0.634 [a] |
| | Incomplete bowel evacuation sensation | 53(34.2) | 29(29.3) | 0.415 [a] |
| | Diarrhea | 71(45.8) | 44(44.4) | 0.832 [a] |
| | Abdominal pain relieved by defecation | 72(46.5) | 45(45.5) | 0.876 [a] |
| | Pollakiuria | 32(20.6) | 30(30.3) | 0.081 [a] |
| | Cold sweat | 59(38.1) | 44(44.4) | 0.317 [a] |
| | Pale face | 65(41.9) | 41(41.4) | 0.935 [a] |

| | | | | |
|---|---|---|---|---|
| | Low back pain | 133(85.8) | 88(88.9) | 0.476 [a] |
| | Dysmenorrhea | 126(81.3) | 74(74.7) | 0.214 [a] |
| Lifestyle | | | | |
| Smoke | Never | 154(99.4) | 95(96.0) | 0.057 [b] |
| | Sometimes | 1(0.6) | 3(3.0) | |
| | Always | 0(0.0) | 1(1.0) | |
| | Everyday | 0(0.0) | 0(0.0) | |
| Alcohol | Never | 92(59.4) | 61(61.6) | 0.720 [b] |
| | Sometimes | 63(40.6) | 38(38.4) | |
| | Always | 0(0.0) | 0(0.0) | |
| | Everyday | 0(0.0) | 0(0.0) | |
| Exercise | Never | 23(14.8) | 19(19.2) | 0.656 [b] |
| | Sometimes | 117(75.5) | 63(63.6) | |
| | Always | 15(9.7) | 15(15.2) | |
| | Everyday | 0(0.0) | 2(2.0) | |
| Coffee | Never | 58(37.4) | 39(39.4) | 0.753 [b] |
| | Sometimes | 82(52.9) | 45(45.5) | |
| | Always | 13(8.4) | 8(8.1) | |
| | Everyday | 2(1.3) | 7(7.1) | |
| Tea | Never | 37(23.9) | 32(32.3) | 0.591 [b] |
| | Sometimes | 103(66.5) | 52(52.5) | |
| | Always | 11(7.1) | 11(11.1) | |
| | Everyday | 4(2.6) | 4(4.0) | |
| Sugary beverage | Never | 8(5.2) | 8(8.1) | 0.986 [b] |
| | Sometimes | 102(65.8) | 61(61.6) | |
| | Always | 44(28.4) | 26(26.3) | |
| | Everyday | 1(0.6) | 4(4.0) | |
| Skipping breakfast | Never | 33(21.3) | 26(26.3) | 0.185 [b] |
| | Sometimes | 70(45.2) | 47(47.5) | |
| | Always | 43(27.7) | 22(22.2) | |
| | Everyday | 9(5.8) | 4(4.0) | |
| Difficulty falling asleep | Never | 27(17.4) | 9(9.1) | 0.630 [b] |
| | Sometimes | 79(51.0) | 63(63.6) | |
| | Always | 39(25.2) | 18(18.2) | |
| | Everyday | 10(6.5) | 9(9.1) | |
| Dreaminess | Never | 9(5.8) | 7(7.1) | 0.773 [b] |
| | Sometimes | 82(52.9) | 49(49.5) | |
| | Always | 53(34.2) | 34(34.3) | |
| | Everyday | 11(7.1) | 9(9.1) | |
| Early awakening | Never | 19(12.3) | 22(22.2) | 0.385 [b] |
| | Sometimes | 75(48.4) | 39(39.4) | |
| | Always | 50(32.3) | 29(29.3) | |
| | Everyday | 11(7.1) | 9(9.1) | |
| Daytime drowsiness | Never | 8(5.2) | 10(10.1) | 0.154 [b] |
| | Sometimes | 75(48.4) | 53(53.5) | |
| | Always | 65(41.9) | 27(27.3) | |

| | | | | |
|---|---|---|---|---|
| | Everyday | 7(4.5) | 9(9.1) | |
| Sleep hour per day | <6 | 150(96.8) | 24(24.2) | 0.463 [a] |
| | ≥6 | 5(3.2) | 75(75.8) | |
| Sit hour per day | <4 | 71(45.8) | 46(46.5) | 0.918 [a] |
| | ≥4 | 84(54.2) | 53(53.5) | |

Footnote:

a Chi-squire; b Mann-Whitney U test; c Fisher exact test;

d Other locations: locations except for parietal, temporal, and frontal;

e Location number: location number defines the same location of the bilateral head as single, different location as multiple, representing the diversity of affected head location;

f Constellation: constellation was classified into yin and yang constellation, yin and yang are traditional Chinese concepts, yin represents negative, inactive, downward, cold and similar features, yang is the opposite, a yin constellation follows a yang constellation and follows another yin constellation when the 12 constellations are arranged in order. specifically, yin constellation includes Taurus, Cancer, Virgo, Scorpio, Capricorn and Pisces, yang constellation includes the rest 6 ones;

g Menstruation regularity and Menstruation length: menstruation regularity and length follow the guidelines for the diagnoses and treatment of abnormal uterine bleeding, regularity refers to variation between menstrual cycle length less than 7 days in the last year, and normal length refers to the length of menstrual bleeding between 3-7 days.

h Perimenstrual symptom number: the number refers to how many perimenstrual symptoms in 27 surveyed ones, specifically had certain symptom between 7 days before menstruation and the end of menstruation in the last year, these symptoms were adopted from clinical researches and guidelines on premenstrual syndrome, but the surveyed items are not enough to give a diagnose of premenstrual syndrome for concession to surveying as many symptoms as possible, so we call them perimenstrual symptoms
