# Supplementary material for: The role of purity and frequency in the classification of perimenstrual headache
Source: BMC Neurol. 2023 Jun 6;23:218. doi: 10.1186/s12883-023-03268-6 (PMC10242800; doi:10.1186/s12883-023-03268-6)
Supplement: Supplementary file 2 — Additional file 2: Table S2. Comparison between impure and pure perimenstrual headache (variables with no statistical difference). [file 12883_2023_3268_MOESM2_ESM.pdf]

TableS2 Comparison between impure and pure perimenstrual headache (variables with no statistical difference)

		N(%) OR Median(IQR)		p
		Impure perimenstrual headache N=147	Pure perimenstrual headache N=107	
Headache feature				
Headache duration	Shorter than 4h	31(21.1)	26(24.3)	0.545 [a]
	Longer than 4h	116(78.9)	81(75.7)	
Headache side	Unilateral	75(51.0)	47(43.9)	0.264 [a]
	Bilateral	72(49.0)	60(56.1)	
Parietal	No	95(64.6)	73(68.2)	0.550 [a]
	Yes	52(35.4)	34(31.8)	
Frontal	No	89(60.5)	75(70.1)	0.116 [a]
	Yes	58(39.5)	32(29.9)	
Temporal	No	40(27.2)	42(39.3)	0.403 [a]
	Yes	107(72.8)	65(60.7)	
Other location [d]	No	85(57.8)	74(69.2)	0.065 [a]
	yes	62(42.2)	33(30.8)	
Throbbing headache	No	81(55.1)	71(66.4)	0.071 [a]
	Yes	66(44.9)	36(33.6)	
Accompanying symptom	No	27(18.4)	27(25.2)	0.187 [a]
	Yes	120(81.6)	80(74.8)	
Nausea	No	76(51.7)	53(49.5)	0.733 [a]
	Yes	71(48.3)	54(50.5)	
Vomiting	No	118(80.3)	86(80.4)	0.984 [a]
	Yes	29(19.7)	21(19.6)	
Photophobia	No	125(85.0)	96(89.7)	0.273 [a]
	Yes	22(15.0)	11(10.3)	
Phonophobia	No	65(44.2)	60(56.1)	0.062 [a]
	Yes	82(55.8)	47(43.9)	
Aggravation with activity	No	117(79.6)	90(84.1)	0.106 [a]
	yes	30(20.4)	17(15.9)	
Need pain killer	No	101(68.7)	63(58.9)	0.360 [a]
	Yes	46(31.3)	44(41.1)	
Family history	No	105(71.4)	86(80.4)	0.103 [a]
	Yes	42(28.6)	21(19.6)	
Migraine	No	112(76.2)	80(74.8)	0.794 [a]
	Yes	35(23.8)	27(25.2)	
Demographics				
Age		30(25,34)	29(25,33)	0.369 [b]
BMI		21.3(19.5,23.4)	21.0(19.5,22.6)	0.485 [b]
Ethnicity	Han	137(93.2)	102(95.3)	0.477 [a]
	Not Han	10(6.8)	5(4.7)	
Marital status	Unmarried	74(50.3)	57(53.3)	0.644 [a]
	Others	73(49.7)	50(46.7)	
Constellation [e]	Yang	80(54.4)	50(46.7)	0.266 [a]
	Yin	67(45.6)	57(53.3)	

Education	Below college	44(29.9)	33(30.8)	0.876 [a]
	College and above	103(70.1)	74(69.2)	
Occupation				
Working years		8.0(3.0,13.0)	6.0(3.0,11.0)	0.372 [b]
Department	Internal medicine	89(60.5)	58(54.2)	0.565 [a]
	Surgery department	42(28.6)	34(31.8)	
	Others	16(10.9)	15(14.0)	
Title	Primary nurse	42(28.6)	34(31.8)	0.268 [a]
	Senior nurse	54(36.7)	46(43.0)	
	Nurse in charge and above	51(34.7)	27(25.2)	
Night shift times per month		5.0(1.0,8.0)	4.0(0.0,8.0)	0.874 [b]
Night shift hours per month		16.0(6.0,50.0)	27.0(0.0,50.0)	0.826 [b]
Menstruation				
Menarche age		13(13,14)	13(12,14)	0.408 [b]
Menstruation regularity [f]	Regular	76(51.7)	56(52.3)	0.920 [a]
	Not regular	71(48.3)	51(47.7)	
Menstruation length [f]	Normal	143(97.3)	101(94.4)	0.243 [c]
	Too short or too long	4(2.7)	6(5.6)	
Perimenstrual symptom number [g]		13(9,18)	13(8,19)	0.634 [b]
Perimenstrual symptoms	Irritability	122(83.0)	83(77.6)	0.279 [a]
	Anxiety	104(70.7)	70(65.4)	0.367 [a]
	Depressed mood	77(52.4)	52(48.6)	0.552 [a]
	Fidgeting	55(37.4)	46(43.0)	0.370 [a]
	Social withdrawl	58(39.5)	50(46.7)	0.247 [a]
	Confusion	66(44.9)	50(46.7)	0.772 [a]
	Low concentration	96(65.3)	65(60.7)	0.457 [a]
	Somnolence	63(42.9)	51(47.7)	0.447 [a]
	Insomnia	58(39.5)	52(48.6)	0.147 [a]
	Breast tenderness	111(75.5)	79(73.8)	0.761 [a]
	Swelling of extremities	41(27.9)	33(30.8)	0.609 [a]
	Dizziness	91(61.9)	66(61.7)	0.971 [a]
	Nausea	66(44.9)	51(47.7)	0.662 [a]
	Vomiting	32(21.8)	32(29.9)	0.140 [a]
	Decreased appetite	86(58.5)	60(56.1)	0.699 [a]
	Abdominal bloating	90(61.2)	67(62.6)	0.822 [a]
	Constipation	48(32.7)	37(34.6)	0.748 [a]
	Urgency to defecate	47(32.0)	37(34.6)	0.663 [a]
	Incomplete bowel evacuation sensation	45(30.6)	37(34.6)	0.504 [a]
	Diarrhea	68(46.3)	47(43.9)	0.712 [a]
	Abdominal pain relieved by defecation	70(47.6)	47(43.9)	0.560 [a]
	Pollakiuria	33(22.4)	29(27.1)	0.394 [a]
	Cold sweat	54(36.7)	49(45.8)	0.146 [a]

	Pale face	61(41.5)	45(42.1)	0.929 [a]
	Low back pain	127(86.4)	94(87.9)	0.733 [a]
	Generalized pain	59(40.1)	49(45.8)	0.368 [a]
	Dysmenorrhea	118(80.3)	82(76.6)	0.484 [a]
Life style				
Smoke	Never	144(98.0)	105(98.1)	0.931 [b]
	Sometimes	3(2.0)	1(0.9)	
	Always	0(0.0)	1(0.9)	
	Everyday	0(0.0)	0(0.0)	
Alcohol	Never	82(55.8)	71(66.4)	0.090 [b]
	Sometimes	65(44.2)	36(33.6)	
	Always	0(0.0)	0(0.0)	
	Everyday	0(0.0)	0(0.0)	
Exercise	Never	28(19.0)	14(13.1)	0.312 [b]
	Sometimes	101(68.7)	79(73.8)	
	Always	17(11.6)	13(12.1)	
	Everyday	1(0.7)	1(0.9)	
Coffee	Never	53(36.1)	44(41.1)	0.636 [b]
	Sometimes	78(53.1)	49(45.8)	
	Always	11(7.5)	10(9.3)	
	Everyday	5(3.4)	4(3.7)	
Tea	Never	38(25.9)	31(29.0)	0.683 [b]
	Sometimes	92(62.6)	63(58.9)	
	Always	11(7.5)	11(10.3)	
	Everyday	6(4.1)	2(1.9)	
Sugary beverage	Never	8(5.4)	8(7.5)	0.158 [b]
	Sometimes	91(61.9)	72(67.3)	
	Always	44(29.9)	26(24.3)	
	Everyday	4(2.7)	1(0.9)	
Skipping breakfast	Never	33(22.4)	26(24.3)	0.513 [b]
	Sometimes	65(44.2)	52(48.6)	
	Always	44(29.9)	21(19.6)	
	Everyday	5(3.4)	8(7.5)	
Difficulty falling asleep	Never	17(11.6)	19(17.8)	0.510 [b]
	Sometimes	86(58.5)	56(52.3)	
	Always	33(22.4)	24(22.4)	
	Everyday	11(7.5)	8(7.5)	
Dreaminess	Never	9(6.1)	7(6.5)	0.180 [b]
	Sometimes	71(48.3)	60(56.1)	
	Always	53(36.1)	34(31.8)	
	Everyday	14(9.5)	6(5.6)	
Early awakening	Never	25(17.0)	16(15.0)	0.941 [b]
	Sometimes	64(43.5)	50(46.7)	
	Always	45(30.6)	34(31.8)	
	Everyday	13(8.8)	7(6.5)	
Daytime drowsiness	Never	9(6.1)	9(8.4)	0.441 [b]

	Sometimes	79(53.7)	49(45.8)	
	Always	52(35.4)	40(37.4)	
	Everyday	7(4.8)	9(8.4)	
Sleep hour per day	<6	40(27.2)	29(27.1)	0.985 [a]
	≥6	107(72.8)	78(72.9)	
Sit hour per day	<4	69(46.9)	48(44.9)	0.743 [a]
	≥4	78(53.1)	59(55.1)	

Footnote:

a Chi-squire; b Mann-Whitney U test; c Fisher exact test;

d Other locations: locations except for parietal, temporal, and frontal;

e Constellation: constellation was classified into yin and yang constellation, yin and yang are traditional Chinese concepts, yin represents negative, inactive, downward, cold and similar features, yang is the opposite, a yin constellation follows a yang constellation and follows another yin constellation when the 12 constellations are arranged in order. specifically, yin constellation includes Taurus, Cancer, Virgo, Scorpio, Capricorn and Pisces, yang constellation includes the rest 6 ones;

f Menstruation regularity and Menstruation length: menstruation regularity and length follow the guidelines for the diagnoses and treatment of abnormal uterine bleeding, regularity refers to variation between menstrual cycle length less than 7 days in the last year, and normal length refers to the length of menstrual bleeding between 3-7 days.

g Perimenstrual symptom number: the number refers to how many perimenstrual symptoms in 27 surveyed ones, specifically had certain symptom between 7 days before menstruation and the end of menstruation in the last year, these symptoms were adopted from clinical researches and guidelines on premenstrual syndrome, but the surveyed items are not enough to give a diagnose of premenstrual syndrome for concession to surveying as many symptoms as possible, so we call them perimenstrual symptoms
